# Infrared Video Pupillography Coupled with Smart Phone LED for Measurement of Pupillary Light Reflex

**DOI:** 10.3389/fnint.2017.00006

**Published:** 2017-03-07

**Authors:** Lily Yu-Li Chang, Jason Turuwhenua, Tian Yuan Qu, Joanna M. Black, Monica L. Acosta

**Affiliations:** ^1^School of Optometry and Vision Science, Faculty of Medical and Health Sciences, The University of AucklandAuckland, New Zealand; ^2^Auckland Bioengineering Institute, The University of AucklandAuckland, New Zealand; ^3^Centre for Brain Research, The University of AucklandAuckland, New Zealand; ^4^New Zealand National Eye Centre, The University of AucklandAuckland, New Zealand

**Keywords:** optometry, pupil light reflex, ophthalmology, autonomic nervous system, cholinergic deficiency

## Abstract

Clinical assessment of pupil appearance and pupillary light reflex (PLR) may inform us the integrity of the autonomic nervous system (ANS). Current clinical pupil assessment is limited to qualitative examination, and relies on clinical judgment. Infrared (IR) video pupillography combined with image processing software offer the possibility of recording quantitative parameters. In this study we describe an IR video pupillography set-up intended for human and animal testing. As part of the validation, resting pupil diameter was measured in human subjects using the NeurOptics^™^ (Irvine, CA, USA) pupillometer, to compare against that measured by our IR video pupillography set–up, and PLR was assessed in guinea pigs. The set-up consisted of a smart phone with a light emitting diode (LED) strobe light (0.2 s light ON, 5 s light OFF cycles) as the stimulus and an IR camera to record pupil kinetics. The consensual response was recorded, and the video recording was processed using a custom MATLAB program. The parameters assessed were resting pupil diameter (D1), constriction velocity (CV), percentage constriction ratio, re-dilation velocity (DV) and percentage re-dilation ratio. We report that the IR video pupillography set-up provided comparable results as the NeurOptics^™^ pupillometer in human subjects, and was able to detect larger resting pupil size in juvenile male guinea pigs compared to juvenile female guinea pigs. At juvenile age, male guinea pigs also had stronger pupil kinetics for both pupil constriction and dilation. Furthermore, our IR video pupillography set-up was able to detect an age-specific increase in pupil diameter (female guinea pigs only) and reduction in CV (male and female guinea pigs) as animals developed from juvenile (3 months) to adult age (7 months). This technique demonstrated accurate and quantitative assessment of pupil parameters, and may provide the foundation for further development of an integrated system useful for clinical applications.

## Introduction

The pupillary light reflex (PLR) is a unique physiological mechanism that allows the examination of the function of the autonomic nervous system (ANS) gated by a light stimulus (McLeod and Tuck, 1987; Neuhuber and Schrödl, 2011; Kaiser et al., 2014; McDougal and Gamlin, 2015). Pupil assessment has been proposed in the evaluation of cholinergic deficiency syndromes such as Alzheimer’s disease and Parkinson’s disease (Takagi et al., 1999; Granholm et al., 2003; Fotiou et al., 2009; Chang et al., 2014). An abnormal PLR may also be suggestive of degeneration or lesion in the ANS (Arendt et al., 1983; Borson et al., 1989; Scinto et al., 2001). Examples of abnormal pupil reflex resulting from neurological problems includes Adie’s tonic pupil, Argyll Robertson Pupil and Horner’s syndrome. Furthermore, dilated pupils that are unresponsive or sluggish are indicative of medical conditions such as oculomotor nerve palsy, trauma, or inflammation (Caglayan et al., 2013). In a clinical setting PLR is examined by observation of the direct response elicited by light in the ipsilateral eye, and consensual response of the contralateral eye (Kaiser et al., 2014). Other relevant parameters examined include resting pupil size, and the relative afferent pupillary response (Kaiser et al., 2014). These observations are made with the use of a bright pen torch shone into the eyes, rely heavily on the judgment of an experienced clinician, and provide qualitative information only. Commercially available pupillometers are useful clinical tools that measure pupil diameter by first capturing an image of the eye, followed by quantification of the pupil diameter in the static image. However, this type of measurement is a lost opportunity to record the dynamic rate and magnitude of pupil diameter change over time. Similarly, pupil measurements in animals have been described in the literature (Pennesi et al., 1998; Dabisch et al., 2008; Mohan et al., 2013; Ostrin et al., 2014) but the parameters measured were limited to pupil size by the experimental set up or by the method of data analysis. A more informative and quantitative way of examining the pupils may be achieved by infrared (IR) videography, that allows the pupil constriction velocity (CV), re-dilation velocity (DV), pre and post illumination pupil sizes to be calculated (Miki et al., 2008; Mohan et al., 2013; Kaiser et al., 2014).

In recent years it has become increasingly common to utilize smart devices in ocular assessment due to their portability, ease of access, and versatility in the adjustment of color and brightness display. Currently smart phones are used to assess color vision, astigmatism, pupil size, macula integrity by the Amsler grid test, and fundus photography (Busis, 2010; Bastawrous, 2012; Bastawrous et al., 2012). Hence, an IR videography set-up coupled with a smart phone application seems an accessible approach in the assessment of the PLR in the clinical setting (Fotiou et al., 2000; Busis, 2010; Giza et al., 2011; Mohan et al., 2013). Such quantitative method is also more informative than the conventional technique using a pen torch, as the information could be used for future analysis and comparison.

We describe in this article the development of a stepwise protocol for an IR video pupillography set-up. This study was inspired by our previously published review article (Chang et al., 2014), which described abnormal pupil reflex in patients with Alzheimer’s disease. Our goal is to optimize an IR video pupillography set-up, which will be used to accurately assess pupil reflex in human and animal models of Alzheimer’s disease. We took into consideration the methods of original research articles that assessed human (Fotiou et al., 2000, 2009) and rodent pupils (Taylor et al., 2008; Mohan et al., 2013) to help us determine the illumination sources, video recording system and dark-adaptation period. To validate our IR video pupillography set-up, resting pupil diameter was measured in human subjects, and PLR was studied in guinea pigs, using a protocol that allows quantitative evaluation of the constriction and re-dilation response over time.

## Material and Equipments

### IR Pupillography Validation—Resting Pupil Diameter in Human Subjects

- Pupillometer (model: 59002, Neuroptics Inc., Irvine, CA, USA)
- Infrared (IR) illumination system (5 × 5 mm IR LEDs with peak spectral wavelength 940 nm)
- Clamp stand to hold IR illumination
- Firefly USB 2.0 IR camera (Point Grey Research, Canada)
- Camera tripod/stand
- Point grey FlyCapture program
- Slit lamp biomicroscope (Haag-Streit Diagnostics, Switzerland), or equivalent
- Laptop computer

### IR Pupillography Set-Up—Guinea Pigs

- Infrared illumination system (5 × 5 mm IR LEDs with peak spectral wavelength 940 nm)
- Clamp stand to hold IR illumination
- Firefly USB 2.0 infrared camera (Point Grey Research, Canada)
- Camera tripod/stand
- Smart device with white LED
- Adjustable animal stand
- Point grey FlyCapture program
- Laptop computer

### Personal Protective Equipment—Animal Handling

- Disposable gloves
- Hairnet
- Overshoes
- Scrubs
- Face masks

### Calibration of Light Cycles, Light Source and Data Analysis

- ILT1700 radiometer (International Light Technologies Inc., Peabody, MA, USA)
- iStrobe LED Flash app
- MATLAB

## Stepwise Procedures

### Resting Pupil Diameter in Human Subjects

This part of the study was conducted with approval from the University of Auckland Human Ethics Committee (reference number 010966) and in accordance with the Code of Ethics of the World Medical Association (Declaration of Helsinki). Written informed consent was obtained for experimentation with human subjects.

**Figure 1:**
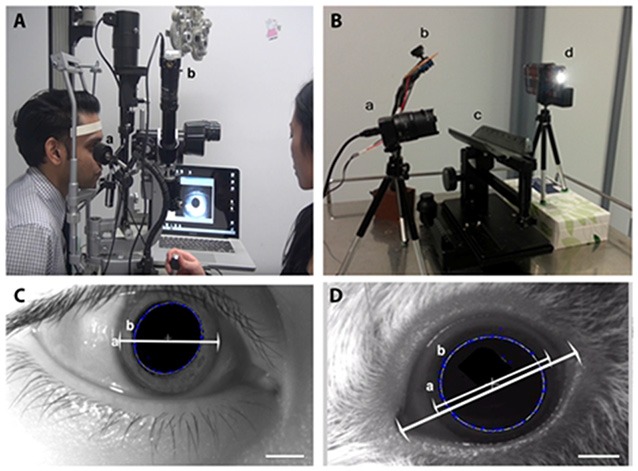
**The infrared (IR) videography set up for pupil diameter measurement.** **(A)** The human set up consists of an IR light emitting diode (LED) for pupil illumination **(a)** attached to a slit lamp biomicroscope for visualization of pupil where the IR camera is attached to an observation tube showing what is seen through the slit lamp eyepiece **(b)** connected to a laptop for video acquisition. Written consent was obtained from Mr. Shashi Patel and we have his permission to show his picture in the demonstration of the procedure conducted by Dr. Lily Chang. **(B)** Guinea pig set up consists of the IR camera on the left **(a)** controlled by a laptop computer, a customized IR LED lighting source **(b)** was placed close to the recorded eye of the animal on the animal stand **(c)** and the flash was delivered by an iPhone flash light **(d)** controlled by a strobelight app (I Strobe). **(C)** Pupil diameter measurement in human using the experimental set-up described in this current study shows the horizontal visible iris diameter **(a)**. The double head arrow shows the extent of the iris diameter and demarcation of pupil margin **(b)**. **(D)** Pupil diameter measurement in guinea pigs and horizontal visible iris diameter **(a)** and the horizontal palpebral aperture length are indicated by the double head arrows. Demarcation of the pupil margin **(b)** is achieved by numerous blue crosses, indicating feature points detected by the program, and join up to be the **(b)** best-fit ellipse. A hexagonal dark patch is created close to the pupil center to remove the corneal reflex from the video.

1. For human subjects our pupillometer (Figure 1A) was set-up by placing an IR illumination system (5 × 5 mm IR LEDs with peak spectral wavelength 940 nm) above the right eye to achieve high contrast filming conditions. A firefly USB 2.0 IR camera (Point Grey Research, Canada) was connected to the laptop computer and the Point grey FlyCapture program was started.
2. The USB 2.0 IR camera was attached to the eye piece of the slit lamp biomicroscope (Haag-Streit Diagnostics, Switzerland), and the focus on the pupil was adjusted by pushing the joystick back and forward.
3. The participant’s horizontal visible iris diameter was measured with the slit beam in the slit lamp to be the scale reference for image analysis (Figure 1C).
4. Room lights and slit lamp were turned off and participant was seated in a dark room and dark adapted for 2 minutes (room light level ≤0.01 cd/m^2^).
5. The participant’s right pupil diameter was measured using a pupillometer (model: 59002, Neuroptics Inc., Irvine, CA, USA).
6. The participant was then seated at the slit lamp biomicroscope and instructed to fixate on a red light on the wall approximately 6 m away. The right pupil was recorded for 10 s in audio visual interleave format using Point grey FlyCapture program at 30 frames per second (fps). Data acquired with the IR camera was compared with the NeurOptics pupillometer measurements.

### Pupillometry Set-Up—Guinea Pigs

This part of the study was conducted according to the University of Auckland Guideline for the experimental use of animals in research approved by the University of Auckland animal research committee (reference number 001138). Guinea pigs (male *n* = 6, female *n* = 5) were 12–28 weeks old. The guinea pigs were bred in open enclosures and in a social group of five or more animals. Water and food was provided *ad libidum*. The light intensity of the breeding room was 1388 watt per steradian for the male enclosure and 1010 watts per steradian in the female enclosure.

1. The pupil recording station was set up for PLR measurement in guinea pigs (Figure 1B). An animal stage was placed in the center of the station with a firefly USB 2.0 IR camera (Model #FMVU-03MTM-CS, 752 × 480 pixels, Point Grey Research, Canada) placed close to the animal’s right eye. An IR illumination system using 5× IR LED with peak spectral wavelength 940 nm rated at 100 mW was also placed close to the right eye to achieve high contrast filming conditions.
2. The USB 2.0 IR camera was connected to the laptop computer and the recording was done via the Point grey FlyCapture program.
3. The smart device strobe light stimulus (spectrum 405–780 nm white light, oval-shaped, 2 × 4.5 mm) was placed on the left side of the animal stage. The light stimulus was set to be seven cycles of 0.2 s light ON—5 s light OFF using the iStrobe LED Flash app (Work Avoidance Ltd). Calibration of the smart device light stimulus intensity was done by ILT1700 radiometer (International Light Technologies Inc., Peabody, MA, USA). The strobe duration of the light stimulus was calibrated by a custom MATLAB program, which detected change in light intensity at each frame of the video.
4. The guinea pig was transported from the animal enclosure to the experimental room. Its right eye horizontal palpebral aperture (distance from temporal to nasal canthus) was measured using a ruler.
5. The guinea pig was gently held on an animal stage and left on the stage for 5 minutes before the procedure to allow familiarization and to adapt to the surrounding and the presence of the researchers. This was followed by a 3 minutes dark adaptation period.
6. The right eye was video-recorded for 10 s in the dark, followed by seven cycles of 0.2 s light ON, 5 s light OFF of the smart device LED illumination. The video was recorded using the FlyCapture Viewer (Point Grey Research) in audio visual interleave format and the videos were analyzed using the custom MATLAB program. The videos were approximately 1 minute long.

### Pupillary Response Video Analysis

A custom MATLAB program (Mathworks; Natick, MA, USA) was used to define the pupil area by a best-fit ellipse, formed by the points where the highest contrast were detected using the starburst edge detection toolbox in MATLAB. The corneal reflection in the video was removed to allow the software to correctly locate the border of the pupil. The pupil center of the first frame was manually selected and the selected points were traced in the successive frame. The horizontal palpebral aperture measured for each guinea pig was used to set the scale in the custom MATLAB program (Figure 1D). A least square ellipse was fitted to the feature points and, 1D median filter with a window size of 10 frames was applied to the raw data to remove sampling noise. The custom MATLAB program outputs a spreadsheet with each frame number corresponding to a pupil diameter value. We assessed the following parameters, using the following equations:

The parameters were calculated using the following equations:

1. Resting pupil diameter = Average across resting period^a^
2. Constriction velocity = Δ diameter/Δ time
3. % Constriction ratio = (Constricted pupil diameter/Resting pupil diameter) × 100
4. Re-dilation velocity = Δ diameter/Δ time
5. % Re-dilation ratio = (Re-dilated pupil diameter^b^/Resting pupil diameter^c^) × 100
  • ^a^Resting period: the amount of time elapsed between the end of dark adaptation to the time point just before the first cycle of pupil stimulation by white LED light.
  • ^b^Re-dilated pupil diameter: the pupil diameter reached after a period of recovery (5 s light OFF) following a light stimulation cycle.
  • ^c^Resting pupil diameter: the pupil diameter measured at the beginning of the videography recording, before stimulation of the pupil by the white LED light.
  • AvCR was the average % Constriction ratio of the 7 light cycles.
  • AvDR was the average % Re-dilation ratio of the 7 light cycles.

### Statistical Analysis

Parameters were statistically analyzed using one-way analysis of variance (ANOVA) of the means of two independent groups for the influence of one categorical unrelated variable (PLR parameters) on one dependent variable (age, gender). *p* < 0.05 was considered statistically significant.

## Anticipated and Representative Results

### Resting Pupil Diameter in Human Subjects

**Figure 2:**
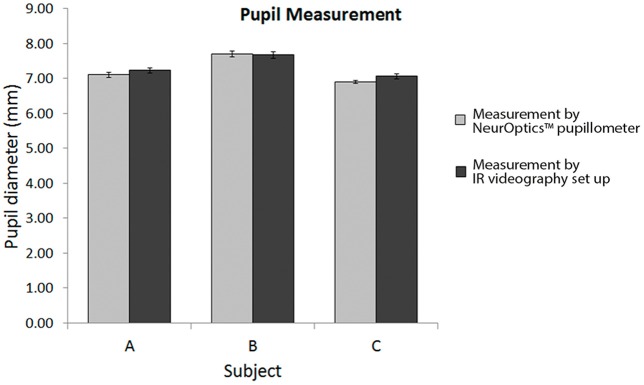
**Comparison of pupil size measurement using NeurOptics pupillometer and the IR videography set up**.

We intended to verify our pupillography set-up by comparing the pupil diameter measurements obtained with a commercially available pupillometer. The horizontal visible iris diameter (measured by the slit lamp, Figure 1C) of each participant was used as a scale bar for image analysis of the IR pupillography video. Repeated measurements of the resting pupil diameter using the NeurOptics^TM^ pupillometer was comparable with the values obtained with our IR video pupillography system in three subjects (Figure 2). The results showed no significant differences between measurements using the NeurOptics^TM^ pupillometer vs. our IR video pupillography (One way ANOVA, *p* > 0.05).

### Pupillometry in Guinea Pigs (PLR)—Calibration of Light Cycles and Light Source

The smart device light emitting diode (LED) light source was placed 5 cm away from the animal’s eye. The intensity of the flash was measured to be 2300 watt per steradian at 5 cm by the ILT1700 radiometer.

**Figure 3:**
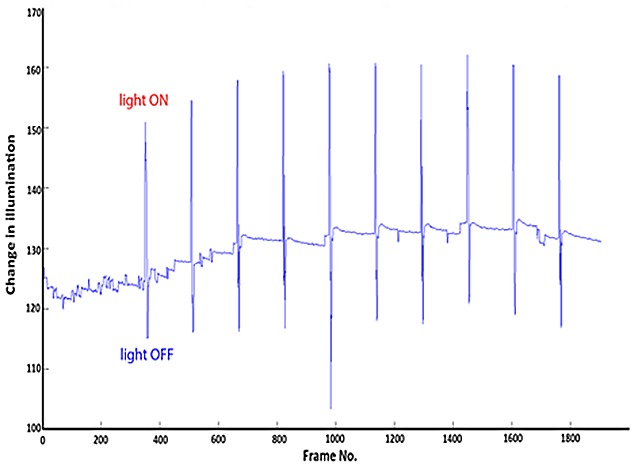
**Change in illumination vs. frame number of the video.** The peaks correlate to light ON and the troughs correlate to light OFF.

The guinea pig video recording was analyzed by the custom MATLAB program, which detected the greatest positive and negative change in illumination. The positive peaks would correlate to the frame numbers when the strobe light turned ON, and the negative troughs correlate to the frame numbers when the strobe light turned OFF (Figure 3). Our results showed the ON duration was 210.00 ± 22.50 ms, and the OFF duration was 5018.52 ± 17.57 ms.

### Pupillometry in Guinea Pigs (PLR)—Representative Results

**Figure 4:**
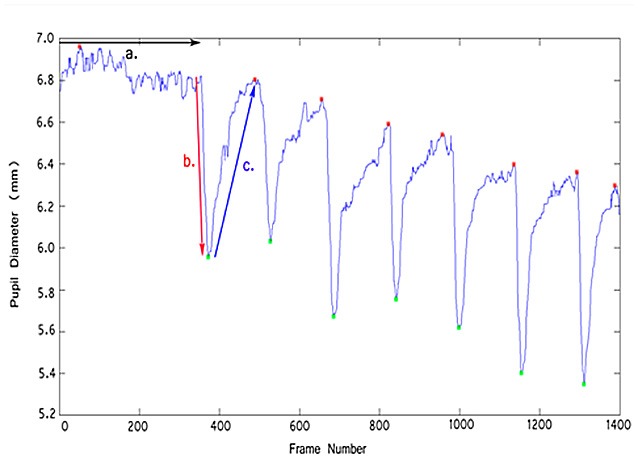
**Change in pupil diameter (mm) vs. frame number of the video.** Resting pupil diameter was recorded over the first 350 frames **(a)**. The pupil was then illuminated by the LED light (light ON) which led to constriction of pupil diameter **(b)**, followed by pupil re-dilation **(c)** during light OFF.

The pupillometry set-up and data output from the MATLAB program yielded the following graph, which shows how pupil diameter changes over the light ON and OFF cycles during video recording (Figure 4). Further data output may be obtained from the graph to calculate the resting pupil diameter, constriction and DV, % constriction and re-dilation ratio as described in stepwise procedures. During protocol development these parameter were assessed at two time points in male and female guinea pigs’ development (at 3 months and 7 months old).

#### PLR Measurement in Guinea Pigs Shows Gender Differences

Male juvenile guinea pigs had larger resting pupil size than female guinea pigs (M = 7.42 ± 0.20 mm vs. F = 5.43 ± 0.32 mm; One way ANOVA, *p* < 0.05; Table 1). In terms of PLR kinetics, all parameters except the average constriction ratio (AvCR) were significantly different between male and female juvenile guinea pigs. Juvenile males had quicker PLR recovery kinetics, greater CV than females (*M* = 2.02 ± 0.18 mm/s vs. *F* = 1.13 ± 0.10 mm/s, *p* < 0.01) and greater DV (*M* = 0.41 ± 0.05 mm vs. *F* = 0.22 ± 0.02 mm, *p* < 0.001). Juvenile males had both quicker pupil CV and DV suggestive of a more readily mobile state of both the sphincter and dilator pupillae on stimulation of a PLR (Table 1). In adult female and male guinea pigs, the AvCR (*p* < 0.05) and average re-dilation ratio (AvDR; *p* < 0.001) were significantly greater in females.

**Table 1:** **Pupillary light reflex (PLR) parameters in males and females at juvenile and adult age**.

	3 month-old			7 month-old		
	Male	Female	*p-value*	Male	Female	*p-value*
D1 (mm)	7.42 ± 0.22	5.43 ± 0.32	0.0326*	7.33 ± 0.41	6.93 ± 0.18	0.392
CV (mm/s)	2.02 ± 0.18	1.13 ± 0.10	0.003**	0.78 ± 0.09	0.88 ± 0.08	0.414
AvCR (%)	83.86 ± 1.30	85.13 ± 0.89	0.625	86.28 ± 0.59	88.65 ± 0.77	0.0256*
DV (mm/s)	0.41 ± 0.05	0.22 ± 0.02	0.0004***	0.19 ± 0.03	0.17 ± 0.01	0.540
AvDR (%)	95.81 ± 0.72	98.54 ± 0.31	0.0001***	95.92 ± 0.63	99.64 ± 0.65	0.0001***

*D1, Resting pupil diameter; CV, Constriction velocity; AvCR, Average constriction ratio; DV, Re-dilation velocity; AvDR, Average re-dilation ratio. One way analysis of variance (ANOVA) comparison between gender at juvenile and adult ages*. Asterisks indicate: *p < 0.05, **p < 0.01, ***p < 0.001.

#### Age Specific Effect in Guinea Pigs

**Figure 5:**
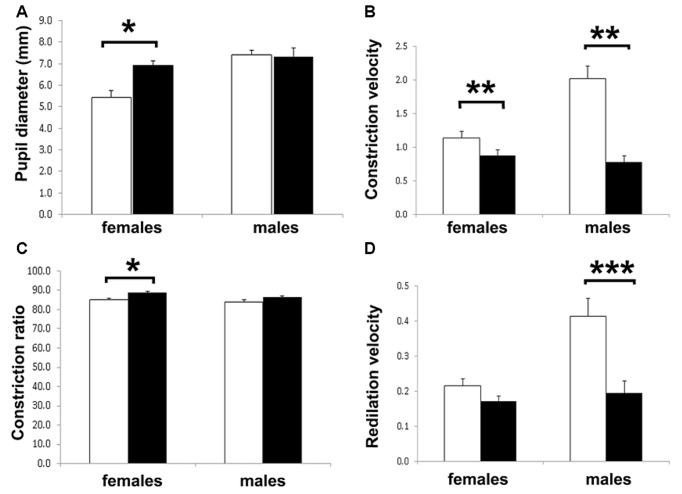
**Pupillary light reflex (PLR) response in juvenile and adult female and male guinea pigs. (A)** Resting pupil diameter was equivalent in males and females except for the younger female age group. **(B)** Constriction velocity (CV) was significantly lower in the adult guinea pigs. **(C)** The constriction ratio was higher only different in the adult female group. **(D)** Re-dilation velocity (DV) showed no differences except for the juvenile female group. Statistical values were obtained using analysis of variance (ANOVA); asterisks indicate: *p < 0.05, **p < 0.01, ***p < 0.001.

There were no significant differences in pupil size between juvenile and adult males; however, juvenile female guinea pigs had significantly smaller pupil size than adults (One way ANOVA, *p* < 0.05; Figure 5A). In terms of PLR kinetics, CV significantly decreased in adult compared with juveniles in both females and males guinea pigs (Figure 5B). AvCR was larger in the adult female group compared with juvenile (*p* < 0.05; Figure 5C), which means adult female pupils were constricting to a lesser extent than juvenile females. This may be suggestive of an age-related decline in parasympathetic input as the mobility of sphincter pupillae was acting more slowly, and constricted to a lesser extent in the adult animals, and that the sympathetic input to the dilator pupillae muscle was relatively more unopposed. Such age-related effect in pupil constriction parameters were not observed in male guinea pigs. DV significantly decreased in males (*p* < 0.001), but was not significant in females (*p* < 0.1; Figure 5D).

## Discussion

The experimental set-up and analysis described in our study is more informative than the ones described in other animal studies (Pennesi et al., 1998; Dabisch et al., 2008; Taylor et al., 2008; Mohan et al., 2013; Ostrin et al., 2014) as we have measured dynamic pupil change over time rather than pupil diameter only. The video pupillography was recorded at a frame rate (30 fps) similar to other animal studies that had sampling rate of every 33 ms, and was sufficient for calculating CV, and DV. Velocity offered more information about the state of the neurons along the visual pathway, as it is a direct indicator of the integrity and strength of the neural signals (Dabisch et al., 2008; Mohan et al., 2013). Our results in female guinea pigs were consistent with that reported by Howlett and McFadden (2007) which described an increase in pupil diameter from 2 days to 200 days of age. Gender-specific pupil data, and parameters such as CV and DV were unavailable in guinea pigs—further investigation is required to confirm the gender and age-specific findings in our anticipated and representative results.

### The Effect of Sampling Rate on IR Video Pupillography

PLR is part of a routine examination for the integrity of the nervous system; it evaluates the state of the autonomic response to light, allowing us to see how efficiently the sphincter pupillae reacts, and how well the dilator pupillae counteracts the sphincter pupillae post illumination. Current pupil studies in animals utilize good quality commercial IR video cameras that are capable of recording at up to 60 fps (Taylor et al., 2008; Mohan et al., 2013). The temporal frequencies of the video recording protocol across animal studies are however highly variable, with Taylor et al.’s (2008) study recording at much lower temporal resolution—1 frame every 2 s. The lack of dynamic pupillary data (continuous measure of rate and magnitude of change) in animal studies is a limitation in existing literature, which we investigated in our study. The inclusion of pupil kinetic in our system—the rate of pupil change during the process of constriction and re-dilation is an innovation in pupil reaction studies in animals. Pupil studies in humans often take more kinetic parameters into account, such as PLR latency, CV, acceleration etc. Fotiou et al. (2009) concluded in their study that maximum pupil CV and acceleration were the best predicators in separating subjects with cholinergic deficiency from normal subjects, while baseline pupil diameter and minimum pupil diameter after pupil constriction to light were not significantly different from controls. This implies that the rate of pupil diameter change is a more relevant measure than pupil diameter when evaluating PLR and iris innervation. Fotiou et al.’s (2009) IR video camera recorded at up to 263 fps, and had high quality light sources, one IR source to illuminate the participant’s face (made of a 32 LED array), and one light stimulus stimulating the pupil with diffuse white light flashes (Taylor et al., 2008; Mohan et al., 2013). This set-up is superior to those described in previous animal studies due to its high sampling rate, good quality videos with high contrast due to the bright IR LED illumination, and a bright light stimulus. However, future studies with video pupillography taken at frame rates comparable with human studies will give us insight in how high the frame rate might need to be to achieve optimal pupil mobility measures in animals.

### The Effect of Illumination on IR Video Pupillography

There are limitations to our techniques, and the light source in particular can be improved for future development. One potential drawback of our light stimulus for initiating PLR is that the white LED light of the smart device was a point source, so accurate positioning of the light was necessary to ensure the same amount of light reached the eye across all animals. A LED diffuser lens fitted in front of the smart device LED will ensure that the stimulus can illuminate the pupil more evenly, even if the position of the stimulus alters during experiment. A brighter IR illumination system would allow better illumination of the guinea pig pupil for a higher-contrast video, which would enable our custom MATLAB software to better discriminate pupil margin for data analysis. The IR illumination could also be integrated with the camera—i.e., an array of approximately 20 IR LEDs can be soldered onto an electrical board and fitted around the camera lens by a collar.

## Conclusion

The IR video pupillography technique described in this study is effective, accessible and easy to assemble. It allows measurement of pupil diameter as well as the dynamic rate and magnitude of pupil change over time. We analyzed the PLR response in normal guinea pigs using parameters such as CV and DV, that are more comparable with human data (Fotiou et al., 2009), and may assist in transitional research looking at both animal models and human participants. Our IR video pupillography set-up can be applied to clinical research in human, as well as in animal models of Parkinson disease and Alzheimer’s disease, that are known to have cholinergic deficits. PLR is becoming an increasingly popular tool in neurological and eye research, contributing to the examination of the ANS, and the retina and optic nerve of the eye. The experimental set-up described in this study may provide a foundation for further development of a more integrated system, which can be used in research as well as in ophthalmological assessments in the clinical setting.

## Author Contributions

LY-LC, MLA and JT designed the study. JT and TYQ designed the MATLAB protocols. JMB proofread and discussed the article. LY-LC, MLA and TYQ conducted the experiments and analyzed the data. LY-LC and MLA prepared the manuscript.

## Conflict of Interest Statement

The authors declare that the research was conducted in the absence of any commercial or financial relationships that could be construed as a potential conflict of interest.

## Abbreviations

ANS: autonomic nervous system
IR: infrared
LED: light emitting diode
PLR: pupillary light reflex.
